# Effect of Molten Pool Size on Microstructure and Tensile Properties of Wire Arc Additive Manufacturing of Ti-6Al-4V Alloy

**DOI:** 10.3390/ma10070749

**Published:** 2017-07-04

**Authors:** Qianru Wu, Jiping Lu, Changmeng Liu, Hongli Fan, Xuezhi Shi, Jie Fu, Shuyuan Ma

**Affiliations:** School of Mechanical Engineering, Beijing Institute of Technology, Beijing 100081, China; 2120140380@bit.edu.cn (Q.W.); jipinglu@bit.edu.cn (J.L.); fanhongli2000@sina.com (H.F.); shixuezhisheo@gmail.com (X.S.); bitfujie@gmail.com (J.F.); bitmc@bit.edu.cn (S.M.)

**Keywords:** wire arc additive manufacturing, Ti-6AL-4V, molten pool size, microstructure, tensile properties

## Abstract

Wire arc additive manufacturing (WAAM) technique is a cost-competitive and efficient technology to produce large structure components in industry domains. Mechanical properties are mainly dominated by the microstructure of the components, which is deeply affected by the molten pool size. In this work, to investigate the effect of the molten pool size on microstructure and mechanical properties of the components, a series of Ti-6Al-4V alloy blocks with different width of molten pool (WMP) ranging from 7 mm to 22 mm were deposited by adjusting the wire feed speed (WFS) from 100 cm/min to 500 cm/min. It is interesting to find that the macrostructure changes from columnar grains to equiaxial grains, and then returns to large columnar grains with the increase of WMP, which is mainly caused by the different cooling rates and thermal gradients. Nonetheless, the tensile properties of the components have a tendency to decline with the increase of WMP.

## 1. Introduction

Additive manufacturing (AM) is a relatively novel concept, including laser additive manufacturing (LAM), electron beam additive manufacturing (EBAM) and wire arc additive manufacturing (WAAM). In this way, parts or components are fabricated by adding material in the form of powder or wire in successive layers [1,2,3,4]. AM can significantly reduce the time between ideal concept and actual part fabrication and produce components at a very low buy-to-fly ratio, contributing to the great popularity in aerospace, automobile, medical and other domains [5,6,7].

**Figure 1:**
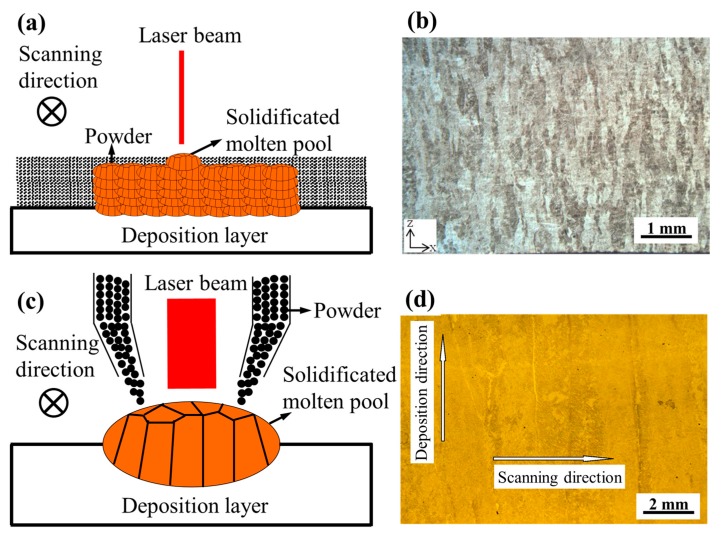
Schematic of selective laser melting (SLM) process (⊗ represents that the scanning direction is perpendicular into the plane.); (**b**) Microstructure of Ti-6Al-4V alloy by SLM [10]; (**c**) Schematic of laser melting deposition (LMD) process and (**d**) Microstructure of Ti-6Al-4V alloy by LMD [11].

Mechanical properties, which are dominated by the microstructures of the components, are the most crucial influencing factors in practical application [8,9]. In additive manufacturing process, components are formed by the “micro molten pool” point by point. Consequently, the microstructure of the components is closely related to the solidification of the “micro molten pool”. In particular, the cooling rate and thermal gradient are greatly influenced by the size of the molten pool. For instance, in selective laser melting (SLM) process, as shown in Figure 1a, a small molten pool size about 0.1 mm can be obtained, resulting in a large cooling rate. The microstructures exhibit fine short columnar β grains under such condition [8,10] (Figure 1b), and the tensile properties of components fabricated by SLM are relatively high. On the contrary, in the laser melting deposition (LMD) process in Figure 1c, the molten pool size is much larger, about 3–10 mm. Compared with the components fabricated by SLM, the mechanical properties of the components is relatively poor, with large coarse columnar β grains (2 mm) microstructures, as shown in Figure 1d [11].

However, the molten pool size is usually large in powder or wire feeding additive manufacturing techniques (i.e., LMD, WAAM and EBAM), which are mainly applied to fabricate large structure components due to their high deposition rate. Basically, the deposition rate of SLM is in the order of 2–10 g/min, whereas it is 50–130 g/min for WAAM [6,12]. Nonetheless, under the condition of small fluctuation of the molten pool size, the research on the influence of molten pool size on the macrostructure, microstructure and mechanical properties of Ti-6Al-4V samples in WAAM has not been reported.

The objective of this study is to investigate the influence of the molten pool size in gas tungsten arc welding (GTAW) based WAAM technique. Five different block samples were fabricated under different processing parameters. In addition, finite-element-method simulations of the additive manufacturing process were performed, as well to additionally study the effect of thermal cycle on the deposition block with different molten pool sizes. Through this study, components with good mechanical properties can be obtained with proper molten pool size, which is helpful to promote the process optimization in the future.

## 2. Experimental Procedures

### 2.1. Experimental Setup and Manufacturing Process

**Figure 2:**
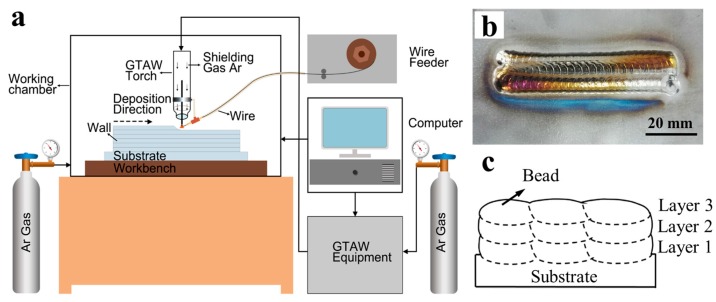
(**a**) Schematic of the experimental setup developed for wire arc additive manufacturing (WAAM) [9]; (**b**) Sample manufactured by WAAM; (**c**) Schematic of cross sections of the samples.

The experimental setup for WAAM is schematically shown in Figure 2a. The additive manufacturing process was carried out in the argon shielding atmosphere in an airtight chamber. The GTAW torch can realize moving upwards and downwards and the workbench can move in the horizontal plane at a specified speed. The wire was fed into the chamber through an annular feed pipe. A 1.4 mm diameter Ti-6Al-4V wire was used for the deposition process. The substrates used in the experiments were hot rolled Ti-6Al-4V plates with the dimension of 200 mm × 100 mm × 5 mm, which were treated by mechanical polishing and then fixed on the workbench before being used.

In the current work, the molten pool size was adjusted by changing the wire feed speed (WFS). The heat input required for per length of the Ti-6Al-4V wire was kept constant (about 21,350 mJ/mm). To investigate the influence of the molten pool size on the macrostructure, microstructures and mechanical properties, five wire arc additive manufactured blocks (80 mm long) were fabricated in this experiment (Figure 2b). All the blocks have three layers, and each layer consists of three beads as indicated in Figure 2c. Low frequency pulse current was applied by the power supply in the experiment. According to careful analysis of previous experiments, optimized deposition parameters were determined (Table 1). There was no preheating during the whole deposition process and the sample was allowed to cool to the same temperature (70 °C) before each new layer was deposited.

### 2.2. Characterization

Two types of samples (for micrographic examination and tensile testing) were cut out from each block. Samples for microstructure observation were cut along building direction including the substrate, and the microstructures were characterized by optical microscopy (Make: Leica, Wetclar, Germany; Model: Leica DM4000M). A series of macro-photographs were taken for each block sample. The resulting image of the entire cross section was used to observe the grain morphology. The macro dimensions and grain dimensions were measured using ImageJ software (https://imagej.net/ImageJ). The samples for micrographic examination were mounted, polished with SiC papers (180, 400, 600, 800, 1000, 1500, 2000 grit) ground and then electrolytic polished using a solution consisting of 60 mL perchloric acid (60 vol %), 390 mL methanol and 350 mL ethylene glycol. All of the micro-images were taken from the second layer of deposited blocks, which is also the position of tensile specimens.

**Figure 3:**
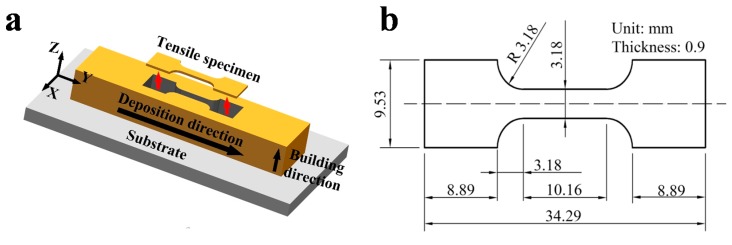
Preparation of tensile samples: (**a**) Manufacturing procedure of tensile specimens; (**b**) Dimensions of tensile specimens.

Tensile specimens were extracted parallel to the deposition plane as shown in Figure 3a. The tensile specimens had a dog-bone shape with a gauge length of 10.16 mm and a 3.18 mm × 0.9 mm cross section [9], as shown in Figure 3b. Over three tensile specimens were taken from each block and the parameters of tensile properties are calculated by averaging the measured data. Tensile tests were carried out with an Instron 5966 electronic universal material testing machine at a strain rate of 9.8 × 10^−4^ s^−1^ at room temperature.

### 2.3. Modeling

**Figure 4:**
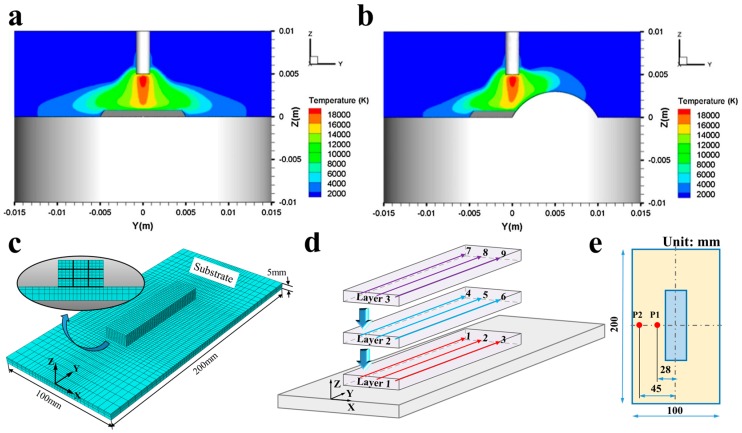
Simulated results of arc temperature of (**a**) single-bead deposition; (**b**) overlapping deposition [13]; (**c**) Three dimensional thermal model and finite element mesh; (**d**) Deposition sequence of the block samples; (**e**) Positions of the two thermocouples on the surface of the substrate.

The finite element software package, ABAQUS (Palo Alto, CA, USA) was used for the thermal model in this research. Early study indicated that the temperature field of the arc would be disturbed by previous deposition in overlapping deposition process, which leads to the asymmetric distribution of the temperature field of the thermal model (Figure 4a,b) [13]. Therefore, the whole material was modelled to accurately reflect the influence of thermal cycle on the deposition block in this study. The height and width of the five deposition block models were measured from the experiments separately. Linear brick elements with 8 nodes (DC3D8) were used for the thermal simulation. In order to capture the high thermal gradients around the heat source during the deposition process, dense meshes were used for the bead and the area near welding line. The meshes became coarser in the ±*x* direction and −*z* direction away from the welding line, which can largely save the calculating time of the model (Figure 4c).

To simulate the material deposition procedure, “element birth technique” was used [14]. All the elements of the deposited block were deactivated at the initial step of the analysis, and then the elements of the nine tracks are activated in turn (Figure 4d) in successive steps to simulate the metal deposition. The user subroutine DFLUX (http://ivt-abaqusdoc.ivt.ntnu.no:2080/v6.14/books/sub/default.htm) in the Fortran code was used to generate the moving heat source for the thermal model. In the current work, the Goldak double ellipsoidal heat source [15,16] was used to apply the heat to the additive manufacturing deposition. All the modelling parameters were identical to the experimental conditions, including the dimension of the models, welding speed, the cooling time between subsequent layers, etc. Thermal properties of the material used in the models were from Zhang and Michaleris [17]. The values of convection coefficient and radiation coefficient were determined by running a series of numerical trials based on the experiments.

To verify the accuracy of the simulation results, the deposition at WFS of 300 mm/min was selected to compare the numerical thermal histories with the experimental thermal histories. Two thermocouples were attached on the surface of the substrate as marked in red (Figure 4e) to record the temperature during the manufacturing process. The predicted temperatures were extracted from the nodal points in the thermal model where the thermocouples were placed in the experiment.

For a given composition, the solidification morphology of the deposited material mainly depends on the velocity of solidification and the thermal gradient [18]. The cooling rate and thermal gradient at the onset of solidification can be extracted from the thermal model results at certain nodal locations. At each nodal location, the solidification cooling rate can be calculated as:

(1) $$\frac{\partial T}{\partial t}=\left|\frac{T_{S}-T_{L}}{t_{S}-t_{L}}\right|$$

where, *T_L_* and *T_S_* represent the liquidus and solidus temperatures reached at times *t_L_* and *t_S_*, respectively. For the material of Ti-6Al-4V used in the current work, the values of *T_L_* and *T_S_* are 1660 °C and 1604 °C, respectively [17,19]. The thermal gradient *G* at the time *t* = *t_L_* is obtained from Fourier’s Law:

(2) $$G=\left|\vec{\nabla }T\right|=\frac{\left|\vec{q}\right|}{k}$$

where $\left|\vec{q}\right|$ represents the magnitude of the heat flux vector and it can be obtained from the simulation results; *k* (34 w m^−1^ °C^−1^ for Ti-6Al-4V) is the thermal conductivity at the liquidus temperature *T_L_*. Then, the solidification velocity *R* can be calculated by the solidification cooling rate and thermal gradient:

(3) $$R=\frac{1}{G}\frac{\partial T}{dt}$$

Following the calculation of *G* and *R*, the expected grain morphology can be predicted as either equiaxed, columnar or mixed by plotting points on the “solidification map” [20].

## 3. Results and Discussion

### 3.1. Macrostructure

**Figure 5:**
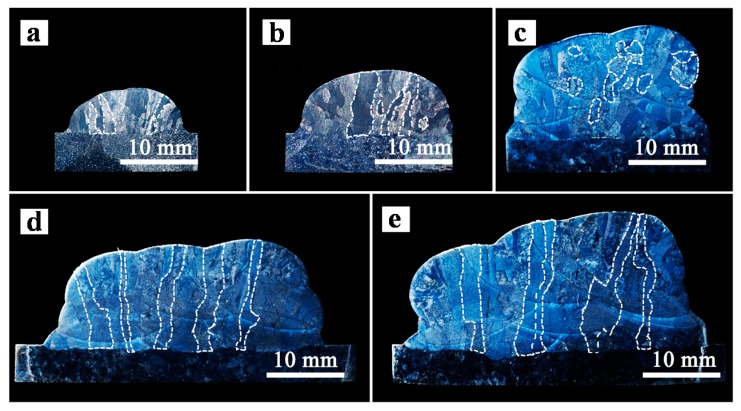
Macroscopic grain morphology of blocks manufactured at the wire feed speed (WFS) of (**a**) 100 cm/min; (**b**) 200 cm/min; (**c**) 300 cm/min; (**d**) 400 cm/min and (**e**) 500 cm/min.

Figure 5 shows the macroscopic grain morphology of specimens manufactured at different WFS. With the increase of WFS, the width and height of the deposition block increases accordingly. The outline of each deposited bead can be seen clearly, indicating the molten pool size, which has a tendency to increase. The width of molten pool (WMP), total width and total height of the deposition, layer thickness and grain width are measured as shown in Table 2. The WMP (7 mm, 10 mm, 14 mm, 19 mm and 22 mm) can be obtained under each WFS condition.

When WMP is 7 mm or 10 mm, the etched cross sections are mainly composed of columnar prior β grains (Figure 5a,b). These grains grow epitaxially and are aligned in the direction of the deposition height across all the three layers, which indicates the steepest thermal gradient direction during manufacturing process. However, more equiaxial grains appear on the cross section of specimens manufactured with WMP of 14 mm. Interestingly, when WMP further increases, the equiaxial grains becomes fewer and the etched cross section exhibits primarily large, columnar grains. The change of the grain morphology with different molten pool sizes is mainly caused by the different cooling rate and solidification rate of the molten pool. Meanwhile, with the increase of WMP, the grain size keeps increasing because of the reducing cooling rate and the grains become much larger and wider (1.23 mm) when WMP is 22 mm.

**Figure 6:**
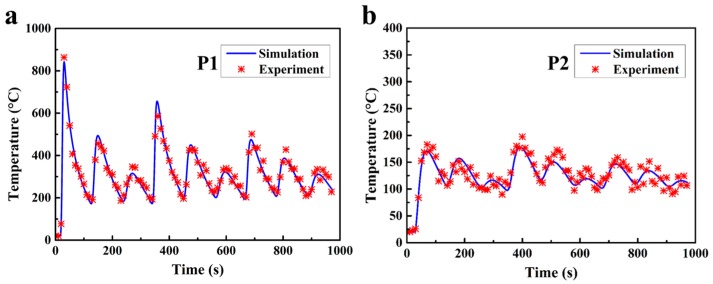
Temperature verification on the measuring positions of (**a**) P1 and (**b**) P2.

Figure 6 shows the comparison between the numerical thermal histories and the experimental thermal histories recorded by the thermocouples at the measuring positions, which are indicated in Figure 4e. Based on the comparison, it was found that the thermal models give relatively accurate predictions of the temperatures at both the thermocouple positions.

**Figure 7:**
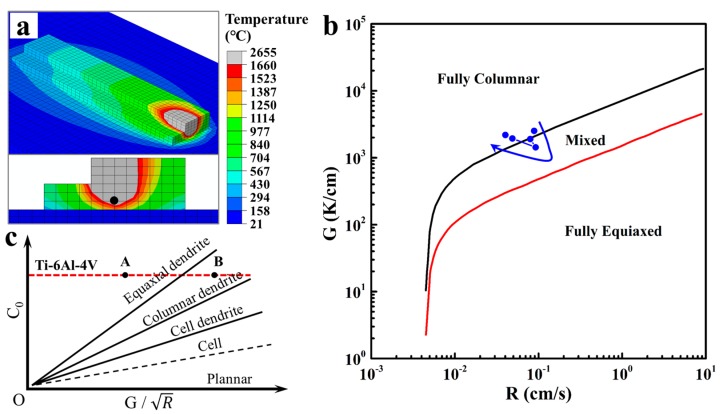
(**a**) Temperature field of deposition blocks; (**b**) Ti-6Al-4V solidification map with simulated data points; (**c**) Effect of $G/\sqrt{R}$ on the grain morphology of solid solution at a specific C_0_ for Ti-6Al-4V.

Figure 7a shows the temperature field of deposition blocks based on finite-element-method simulations. The grey area represents the molten pool, which corresponds with the bead width well. The node near the onset of the solidification is selected to calculate the thermal gradient *G* and solidification velocity *R* (Figure 7a), and the relevant values at different WFS are shown in Table 3. In order to predict the grain morphology of Ti-6Al-4V, the resulting thermal gradient and solidification rate values were plotted on the solidification map [18,21], as shown in Figure 7b. The finite-element-method simulations predicted that the grain morphology has a tendency to change from columnar grains to fully equiaxial grains, and then returns to fully columnar grains with the increase of WMP, which is similar to those observed in the actual cross sections (Figure 5).

According to the numerical simulation results and the observed phenomena during the experiments, the possible reasons of the change of grain morphology at different WMP can be summarized as follows.

**Figure 8:**
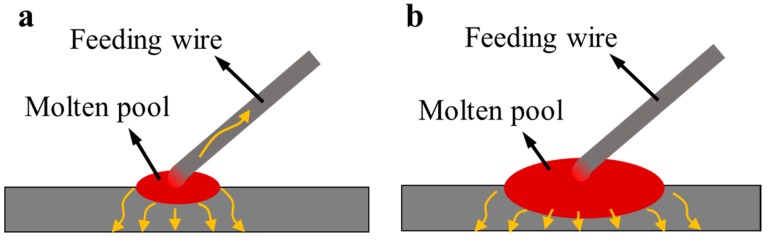
Schematic of heat dissipation at (**a**) small width of molten pool (WMP) and (**b**) large WMP.

When WMP is small (7 mm–14 mm), the size of the feeding wire (1.4 mm in diameter) is relatively large to the molten pool, as indicated in Figure 8a. The wire can take away part of the heat flow. At the WMP of 7 mm and 10 mm, the WFS is relatively slow, namely 100 cm/min and 200 cm/min, respectively. Under this condition, the heat primarily flows down vertically through the substrate, and high thermal gradient (*G*) can be obtained, contributing to the formation of columnar grains. At the WMP of 14 mm, WFS increases to 300 cm/min accordingly, which means more cool wire can be fed into the molten pool. Heat can be largely dissipated by the feeding wire under this condition. Therefore, the high thermal gradient of the molten pool would be disturbed, and the epitaxial growth of the columnar grains from the bottom is restricted [22]. Consequently, more equiaxed grains can be achieved at the WMP of 14 mm.

When WMP further increases (19 mm and 22 mm), the size of the feeding wire is very small to the molten pool (Figure 8b). Compared with the heat dissipation of the substrate, the heat conducted by the wire can be ignored. Heat mainly flows downward through the substrate. With the increase of thermal gradient *G* and decrease of solidification velocity *R* (Figure 7b), the value of $G/\sqrt{R}$ has increased a lot, thus for a given composition of Ti-6Al-4V (a constant value of C_0_), the grains turn to coarse columnar grains with large WMP (Figure 7c, from point A to B). In this case, coarse columnar grain growth is promoted.

### 3.2. Microstructure

**Figure 9:**
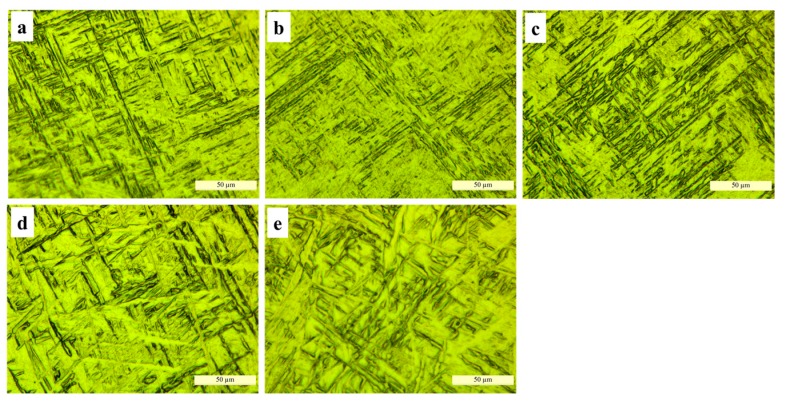
Microstructure of samples manufactured with WMP of (**a**) 7 mm; (**b**) 10 mm; (**c**) 14 mm; (**d**) 19 mm and (**e**) 22 mm.

Representative microstructures from the cross sections in the center of the deposition blocks (layer 2) fabricated with different WMP are shown in Figure 9. The widths of α-lath were observed by analyzing micrographs and measured using ImageJ software. It can be clearly seen that with the increase of WMP, α structure becomes larger and larger.

Figure 9a,b show the long orthogonally oriented α’-martensitic plates with a needle-like morphology, which have been reported in other studies [23]. The α’-lath width are similar in Figure 9a,b. The microstructure difference of Ti-6Al-4V primarily depends on the cooling rate when the β transforms to α since it cools across the transus temperature [8]. At the WMP of 7 mm or 10 mm, the heat input is relatively low and rapid cooling rate can be achieved due to the small size of the molten pool, thus promoting the formation of needle-like α’ phase. At the WMP of 14 mm, the basket weave structures can be found, and the α-lath width is slightly larger (Figure 9c). With the increase of WMP, α structures turn to be coarse lath structures in Figure 9d,e. This is mainly because heat input increases correspondently at larger WMP, whereas the cooling rate drops rapidly, contributing to the increase of α-lath width [24].

### 3.3. Tensile Properties

**Figure 10:**
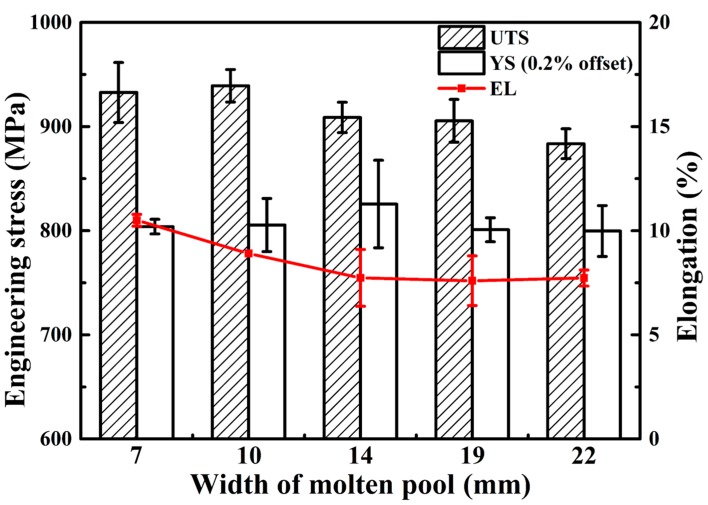
Tensile properties of samples deposited with WMP ranging from 7 mm to 22 mm.

Figure 10 shows the ultimate tensile strength (UTS), yield strength (YS) and the elongation (EL) at failure of tensile specimens derived from the samples fabricated with different WMP. Each group includes over three repeat tests and the last results are averaged. On the whole, with WMP increases, both the strength and elongation at failure have a tendency to decline. The ultimate tensile strength of the tensile specimens ranges from 883 MPa to 940 MPa, yield strength ranges from 800 MPa to 826 MPa, and elongation at failure ranges from 7.6% to 10.5%. These results are primarily attributed to the differences in the macrostructure and microstructure (see Figure 5 and Figure 9).

It has been researched that the strengths (i.e., UTS and YS) are related to both the size of α structure and prior β grains [2]. In this study, with the increase of WMP, the size of the β grains becomes larger (Figure 5) and the α-lath width keeps increasing (Figure 9), contributing to the decreasing strength. Samples manufactured with small WMP (7 mm and 10 mm), composed of needle-like α’ phase (Figure 9a,b), exhibit higher strength than those manufactured at large WMP, which show much coarser α-lath (Figure 9d,e). The declining elongation at failure (red line in Figure 10) is mainly determined by the growing size of the prior β grains [25] (Figure 5). Typically, when WMP reaches 22 mm, the morphology of the cross section has turned into much coarser α-lath within larger prior β grains, leading to very poor strength and elongation performance.

Hence, in the current investigation, the quality of WAAM components manufactured with WMP of 7 mm and 10 mm fulfill at least the minimum requirements for cast Ti-6Al-4V material (elongation at failure of 8% and UTS of 860 MPa, ASTM F1108 [2]). However, only the components manufactured with WMP of 7 mm can meet the requirements for wrought Ti-6Al-4V material (10% and 930 MPa, ASTM F1472 [2]).

## 4. Conclusions

The effect of molten pool size on the microstructure and tensile properties of wire arc additive manufactured Ti-6Al-4V has been systematically investigated in this study. Results from physical experiments and numerical simulations can be used to draw the following conclusions.

1. With the increase of WMP, the macrostructure of Ti-6Al-4V changes from columnar grains (7 mm and 10 mm) to equiaxial grains (14 mm) firstly and then turns into large epitaxial columnar grains (19 mm and 22 mm). The variation of *G* and *R* is plotted on the solidification map of Ti-6Al-4V based on finite-element-method simulations of the additive manufacturing, which shows a similar grain morphology to those observed in the actual sections. It seems that equiaxial grains morphology can be obtained by controlling the cooling rate and thermal gradient through adjusting the manufacturing parameters.
2. With WMP of 7 mm and 10 mm, rapid cooling rate can be obtained. The microstructure of the cross sections turns out to be needle-like α’ phase. However, with larger WMP, the microstructure changes into α lath morphology. With the increase of WMP, α-lath width becomes larger and microstructure turns into coarse α plate morphology due to the increasing heat input with larger WMP.
3. Samples manufactured at small WMP (7 mm and 10 mm) exhibit higher strength than those manufactured at larger WMP. The decrease of elongation at failure can be attributed to the growing size of the prior β grains. Typically, when WMP reaches 22 mm, a very poor strength and elongation performance was observed, because of the much coarser α-lath within larger prior β grains at this time.

## Figures and Tables

**Table 1:** Deposition parameters of WAAM.

No.	WFS, cm/min	Peak Current, A	Hatch Distance, mm	Heat Input, mJ/mm	Welding Speed, mm/min	Base-to-Peak Current Ratio	Peak Time Ratio	Pulse Frequency, Hz	Arc Length, mm
1	100	90	4.2	about 21,350	100	30%	50%	1.2	4
2	200	140	5.1						
3	300	170	7.2						
4	400	210	9.0						
5	500	270	9.8						

**Table 2:** Macroscopic parameters at different WFS.

WFS, cm/min	WMP, mm	Total Width, mm	Total Height, mm	Layer Thickness, mm	Average Grain Width, mm
100	7	14.5	5.7	1.9	0.72
200	10	19.0	8.1	2.7	0.92
300	14	23.1	12.3	4.1	0.98
400	19	32.8	13.5	4.5	1.05
500	22	35.2	16.5	5.5	1.23

**Table 3:** Thermal gradient and solidification velocity of the point at the onset of solidification.

WMP, mm	Thermal Gradient (*G*), k/cm	Solidification Velocity (R), cm/s	$G/\sqrt{R}$, k cm^−1.5^ s^−0.5^
7	2516	0.08868	8449
10	1903	0.07974	6739
14	1431	0.09229	4710
19	1938	0.04866	8786
22	2189	0.04015	10,925
